# Supplementary material for: Complexome profiling on the Chlamydomonas lpa2 mutant reveals insights into PSII biogenesis and new PSII associated proteins
Source: J Exp Bot. 2021 Aug 26;73(1):245–62. doi: 10.1093/jxb/erab390 (PMC8730698; doi:10.1093/jxb/erab390)
Supplement: erab390_suppl_Supplementary_Dataset_S1 [file erab390_suppl_supplementary_dataset_s1.zip › Supplemental Dataset 1 - Excel List and all profiles/plots/PCR1_Cre03.g154550.html]

Trivial name: PCR1  
  
Euclidean distance: 9462.13  
Mean Intensity (WT): 262.29  
Mean Intensity (Mut): 484.06  
Distance: 19.55  
  
MapMan: amino acid metabolism.degradation.glutamate family.arginine;amino acid metabolism.synthesis.glutamate family.proline.pyrroline-5-carboxylate reductase  
  
p value of intensity sums Welch test: 0.3812
